# Requirement for a Uroplakin 3a-Like Protein in the Development of Zebrafish Pronephric Tubule Epithelial Cell Function, Morphogenesis, and Polarity

**DOI:** 10.1371/journal.pone.0041816

**Published:** 2012-07-25

**Authors:** Shalini Mitra, Stefan Lukianov, Wily G. Ruiz, Chiara Cianciolo Cosentino, Subramaniam Sanker, Linton M. Traub, Neil A. Hukriede, Gerard Apodaca

**Affiliations:** 1 Department of Medicine Renal-Electrolyte Division, University of Pittsburgh, Pittsburgh, Pennsylvania, United States of America; 2 Department of Developmental Biology, University of Pittsburgh, Pittsburgh, Pennsylvania, United States of America; 3 Department of Cell Biology, University of Pittsburgh, Pittsburgh, Pennsylvania, United States of America; National Cancer Institute, United States of America

## Abstract

Uroplakin (UP)3a is critical for urinary tract development and function; however, its role in these processes is unknown. We examined the function of the UP3a-like protein Upk3l, which was expressed at the apical surfaces of the epithelial cells that line the pronephric tubules (PTs) of the zebrafish pronephros. Embryos treated with *upk3l*-targeted morpholinos showed decreased pronephros function, which was attributed to defects in PT epithelial cell morphogenesis and polarization including: loss of an apical brush border and associated phospho-ERM proteins, apical redistribution of the basolateral Na^+^/K^+^–ATPase, and altered or diminished expression of the apical polarity complex proteins Prkcz (atypical protein kinase C zeta) and Pard3 (Par3). Upk3l missing its C-terminal cytoplasmic domain or containing mutations in conserved tyrosine or proline residues did not rescue, or only partially rescued the effects of Upk3l depletion. Our studies indicate that Upk3l promotes epithelial polarization and morphogenesis, likely by forming or stimulating interactions with cytoplasmic signaling or polarity proteins, and that defects in this process may underlie the pathology observed in UP3a knockout mice or patients with renal abnormalities that result from altered UP3a expression.

## Introduction

The uroplakins (UP)s are integral membrane proteins originally identified as major constituents of the apical surface of the polarized umbrella cells that form the outermost layer of the uroepithelium [1], [2]. They play an important, but ill-defined role in urinary tract biology and development [3]. Members of the UP family include the tetraspanins UP1a and UP1b, and the single-pass, type I proteins UP2, UP3a, and UP3b. UP3(a/b) is distinct because it is the only UP that has a significant carboxy-terminal cytosolic domain (∼50 amino acids in length). UPs form specific UPIa/UP2 and UP1b/UP3a heterodimers and their oligomerization is a prerequisite for their ER-exit and assembly into the asymmetric unit membrane (AUM) particles that form the plaque regions of the umbrella cell apical membrane [4]. Knockout (KO) mice lacking expression of UP3a have a hyperproliferative epithelium, and their umbrella cells appear undifferentiated, have few plaques, and their apical membrane shows altered permeability to urea and water [5], [6]. These mice also exhibit vesicoureteral reflux and an attendant hydronephrosis [5]; however, there is no apparent genetic link between UP3a and reflux in patients [7]. Intriguingly, *de novo* mutations of UP3a, affecting conserved amino acids in its membrane proximal region and cytoplasmic domain, are found in patients with renal adysplasia [8], [9]. These observations, coupled with the expression of UPs in the urogenital sinus and renal pelvis of the human fetus implicate UPs in proper urinary tract organogenesis [8]. Although the biological function of UP3a within the developmental setting is unclear, recent observations indicate that it can serve as a molecular scaffold that promotes signal transduction during fertilization and upon bacterial infections [10], [11], [12].

To gain further insight into UP3a function during kidney organogenesis we are using zebrafish, which form a functional pronephros that recapitulates the earliest steps in mammalian kidney development [13], [14]. The zebrafish pronephros consists of a pair of segmented pronephric tubules (PTs) joined at their anterior ends by a fused glomerulus and terminate at their posterior ends in the cloaca [14]. The epithelial cells lining the PTs have distinct apical and basolateral domains and renal clearance and maintenance of ionic/osmotic gradients depends on specialized apical membrane domains such as a brush border and cilia, and the polarized distribution and function of ion channels, pumps, and receptors [15], [16]. Intriguingly, the zebrafish genome encodes an UP3a-like protein called Upk3l (as well as a homologs of UP1a and UP2) [17], but its expression, distribution, and function is unknown. We report that Upk3l is expressed at the apical surface of the PT epithelial cells, and that decreased Upk3l expression leads to pronounced defects in pronephros function, likely in response to a failure to form a brush border, mislocalization of the Na^+^/K^+^–ATPase, and altered expression of the apical polarity complex proteins Prkcz (atypical protein kinase C zeta) and Pard3 (par3). Moreover, rescue of these defects was prevented by variants of Upk3l lacking a C-terminal cytoplasmic domain or containing mutations in conserved proline or tyrosine residues. We propose that Upk3l promotes epithelial polarization and morphogenesis during early kidney development and loss of these functions may underlie the defects observed in patients with renal adysplasia resulting from mutated UP3a.

## Results

### Upk3l is expressed at the apical surface of PT epithelial cells

**Figure 1:**
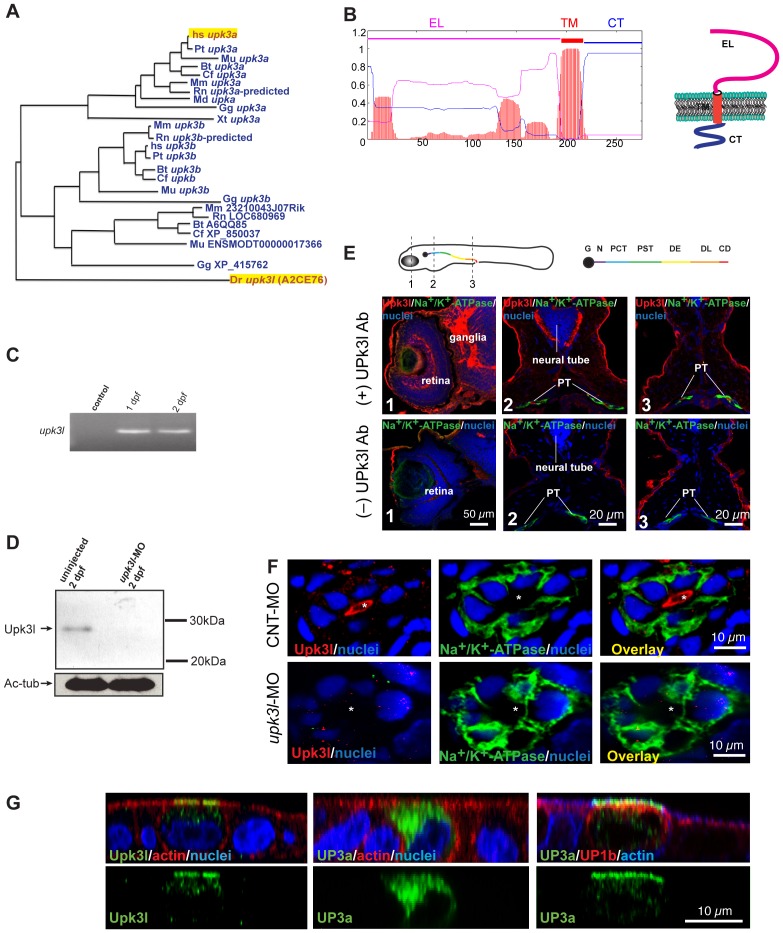
Expression of *upk3l* in the zebrafish pronephros. (A) Dendrogram of the UP3 family (TreeFam accession TF336628) Legend: Bt, *Bos taurus*; Cf, *Canis familiaris*; Dr: *Danio rerio*; Gg, *Gallus gallus*; Hs, *Homo sapiens*; Md, *Monodelphis domestica*; Mm, *Mus musculus*; Mu, *Macaca mulatta*; Pt, *Pan troglodytes*; Rn, *Rattus norvegicus*; Xt, *Xenopus tropicalis*. *Upk3l* (A2CE76) and human (hsUP3a) are highlighted. (B) TMHMM plot and predicted structure of Upk3l: CT, cytosolic tail; EL, extracellular loop; TM, transmembrane domain. (C) Expression of *upk3l* message in 1-dpf and 2-dpf embryos as detected by RT-PCR; control: reaction in which the RNA template was substituted with water. (D) Western blot analysis of Upk3l expression in uninjected embryos or those injected with *upk3l*-MO. Acetylated-tubulin (Ac-tub) was used as a loading control. (E) Localization of Upk3l, Na^+^/K^+^-ATPase, and nuclei in cross sections of 2-dpf embryos. The Upk3l primary antibody was added to the samples in the upper row of images, but excluded from those in the lower set. The approximate location of the sections is shown in the cartoon above. Legend: G, glomerulus; N, neck; PCT, proximal convoluted tubule; PST, proximal straight tubule; DE, distal early tubule; DL, distal late tubule; CD, collecting duct. (F) Distribution of Upk3l, Na^+^/K^+^-ATPase, and nuclei in the PTs of 2-dpf embryos incubated with control MO (upper panels) or *upk3l*-MO (lower panels). The lumen is marked with an asterisk. (G) Immunolabeling of MDCK cells transiently transfected with cDNA encoding *upk3l*, human (h)UP3a alone, or hUP3a and hUP1b. Samples were costained with TRITC-phalloidin to label the cortical actin cytoskeleton and/or TO-PRO-3 to label the nuclei. Images are XZ confocal sections.

Homologs of the UP3a gene are present in a number of species, including zebrafish (Fig. 1A) [17]. TMHMM software predicted that, similar to its mammalian counterpart, the Upk3l protein has an ∼150 amino-acid extracellular loop, an ∼20 amino acid transmembrane domain, and an ∼60 amino acid cytosolic tail domain (CT; Fig. 1B). RT-PCR of cDNA obtained from whole genomic RNA was used to confirm that *upk3l* mRNA was expressed in 1- and 2-day post fertilization (dpf) embryos (Fig. 1C). Likely because of low abundance, we were not able to reliably determine the localization of upk3l mRNA using *in situ* hybridization. Instead, we generated a polyclonal rabbit antibody against residues 245–261 of the predicted Upk3l cytoplasmic domain and used western blot analysis to verify expression of an ∼29 kDa protein in lysates of 2-dpf embryos (Fig. 1D). Morphant embryos, those micro-injected at the 1–2-cell stage with a transcription blocking morpholino (MO) against *upk3l* (*upk3l*-MO), showed reduced levels of Upk3l expression (Fig. 1D). The tissue and subcellular distribution of Upk3l was further examined by immunolabeling frozen sections of 2-dpf embryos. Upk3l staining was observed in the retina of the eye, in ganglia near the eye, and in tissue lateral to the neural tube (possibly ganglia) (Fig. 1E). Upk3l was also expressed along the length of the PTs where it was localized to the apical pole of the lining epithelial cells and was distinct from the Na^+^/K^+^-ATPase, a marker of the basolateral surface (Fig. 1F). Staining for Upk3l was undetectable in the PTs of morphant embryos (Fig. 1F).

When expressed in HEK293T cells [4], or polarized epithelial MDCK cells (Fig. 1G), UP3a does not exit the endoplasmic reticulum without its heterodimerization partner UP1b. However, no UP1b homolog has been reported in the zebrafish genome. To determine whether Upk3l can traffic in the absence of a binding partner, we expressed Upk3l in MDCK cells. Like PTs, exogenously expressed Upk3l was observed at the apical pole of MDCK cells (Fig. 1G), indicating that Upk3l may be able to traffic and function independently of an UP1b homolog.

### *Upk3l* knockdown causes pericardial edema and defective renal clearance

**Figure 2:**
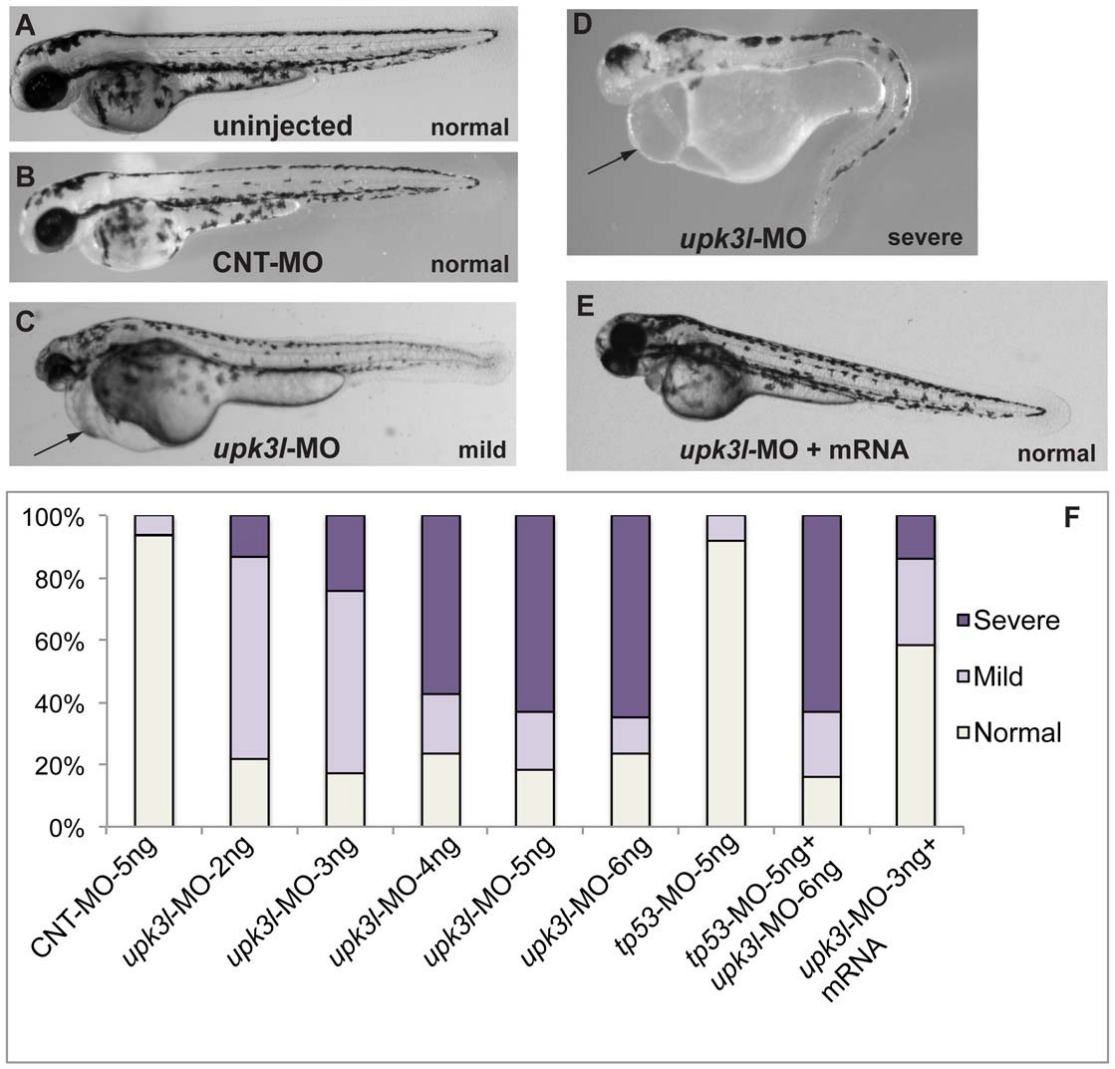
Phenotypes of *upk3l* morphant embryos. (A–D) Uninjected-, CNT-MO-, or *upk3l*-MO-injected embryos were examined under a dissecting microscope and photographed. Pericardial edema, when observed, is indicated by a black arrow. (E) Embryo injected with 3 ng *upk3l*-MO, 100 pg MO-resistant *upk3l* mRNA, and 50 pg of mCherry mRNA (to mark successful injections). Microinjection of MO-resistant mRNA alone at doses of 50–200 pg did not produce detectable phenotypic abnormalities. (F) Distribution of morphological phenotypes associated with embryos injected with 5 ng CNT-MO, 2–6 ng of *upk3l*-MO, *tp56*-MO ± *upk3l*-MO, or *upk3l*-MO/*upk3l* mRNA/mCherry (n = 100).

**Figure 3:**
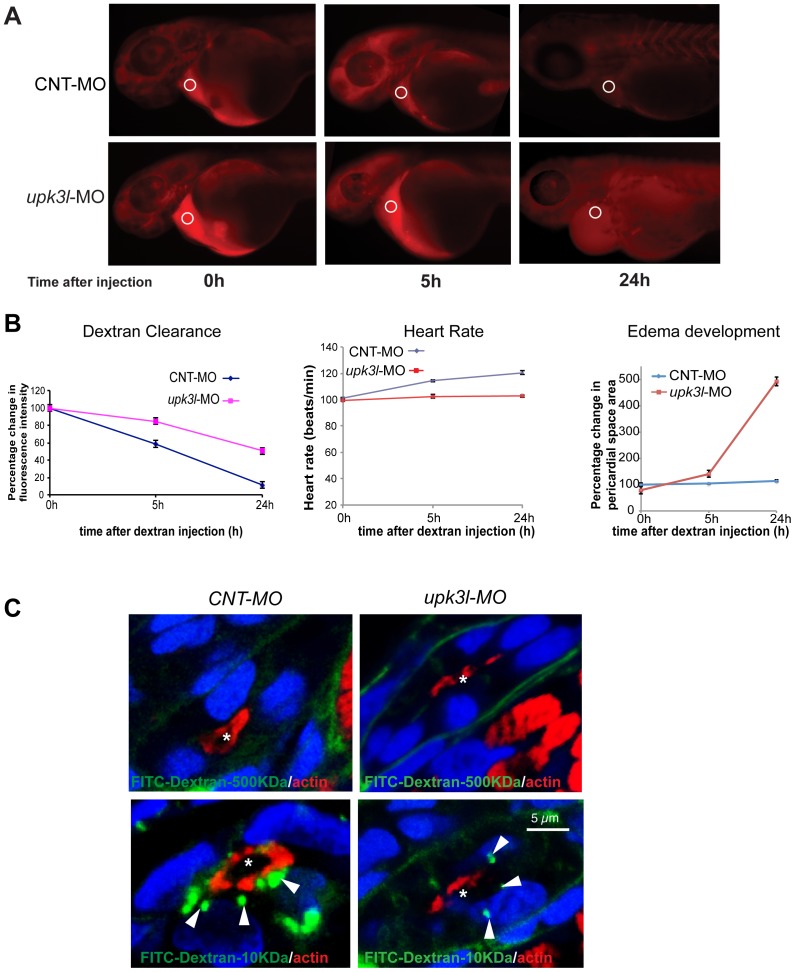
Pronephric clearance, heart rate, and pericardial area in control and morphant embryos. (A) Clearance of 70 kDa-TRITC dextran injected into the common cardinal vein of 54-hpf control (top panel) or morphant (bottom panel) embryos. The fluorescence intensity of the sampled area (circled) was measured in each embryo 0, 5, and 24 h following dextran injection. (B) The heart rate, pericardial area, and the dextran retention was recorded for each embryo and the values relative to t = 0 (post dextran injection) were calculated. Data are reported as mean ± SEM (n = 25). (C) Uptake of 500 kDa or 10 kDa FITC-dextran in control or morphant PT epithelial cells. Tubule lumens are marked by asterisks, while internalized dextran is indicated by arrows. Note the incomplete ring of actin in morphant PT cells.

To explore possible functions of Upk3l, we followed the development of uninjected embryos, those injected with a *upk3l*-specific MO, or those injected with a control MO (CNT-MO) that lacked a specific target or biological activity in zebrafish (Fig. 2A–C). While no gross changes were observed at 2.0 dpf, in 2.5-dpf morphants a pericardial edema (effusion) developed (Fig. 2C–D and Fig. 3B). The edema became more conspicuous by 3 dpf (Fig. 3B). Embryos without pericardial edema and with only minor axis curvature were classified as “normal” (Fig. 2F), whereas those that exhibited pericardial edema but lacked other major phenotypic alterations, other than a small amount of tail-axis curvature, were classified as having a “mild” phenotype (Fig. 2C and F). This was the predominant phenotype observed for embryos injected with low doses of *upk3l*-MO (2–3 ng). However, at higher doses (4–6 ng) a larger percentage of injected embryos had additional defects including loss of circulation, significant tail curvature, and head and eye abnormalities (Fig. 2D and 2F). These were classified as “severe,” and failed to survive beyond 4–5 dpf. Embryos injected with CNT-MO (up to 5 ng) were indistinguishable from uninjected embryos (compare Fig. 2A to 2B), confirming that the observed defects were not caused by the injection procedure. The morphant phenotype was rescued when an MO-resistant form of *upk3l* mRNA was injected into 1-cell stage embryos prior to injection of 3 ng of *upk3l*-MO (Fig. 2E–F). In these embryos, a significant fraction (∼60%) were phenotypically “normal,” verifying that the phenotypic abnormalities observed in morphants resulted specifically from the loss of Upk3l expression. Because MOs can stimulate off-target effects like apoptosis of neuronal cells [18], we examined embryos that were injected singularly with *tp53* (p53)-MO, which prevents MO-induced apoptosis, or embryos co-injected with *tp53*- and *upk3l-MO*. However, embryos injected with *tp53*-MO alone showed a similar phenotype to embryos injected with CNT-MO, and the injection of *tp53*-MOs did not alter the *upk3l* morphant phenotype in any visible manner.

Pericardial edema is often associated with pronephros dysfunction [16], [19], probably because defective electrolyte reabsorption by the PTs leads to fluid imbalance. To examine kidney function we measured renal clearance of 70 kDa TRITC-dextran injected into the common cardinal vein of morphant or control embryos [20]. Neither population of embryos exhibited conspicuous edema at the time of dextran injection. In controls, the majority of dextran was cleared from the circulation (with ∼10% remaining after 24 h) (Fig. 3A–B). In contrast, morphants that developed edema showed a significantly lower dextran clearance, with ∼50% retention after 24 h (Fig. 3A–B). Because renal clearance is contingent on a patent circulatory system, we examined the heart and vasculature of morphant and control embryos derived from a transgenic fish line that expressed the transgene Tg (*fli-1:EGFP*) in endothelial cells [21]. We observed formation of all of the major vessels and the heart (data not shown). Clearance is also dependent on cardiac output, which is a function of heart rate (and stroke volume). However, we observed no detectable change in heart rate during the period of time before edema development, and the heart rate was only decreased by ∼10% in 2.5-dpf morphants and by ∼15% at 3 dpf (Fig. 3B).

**Figure 4:**
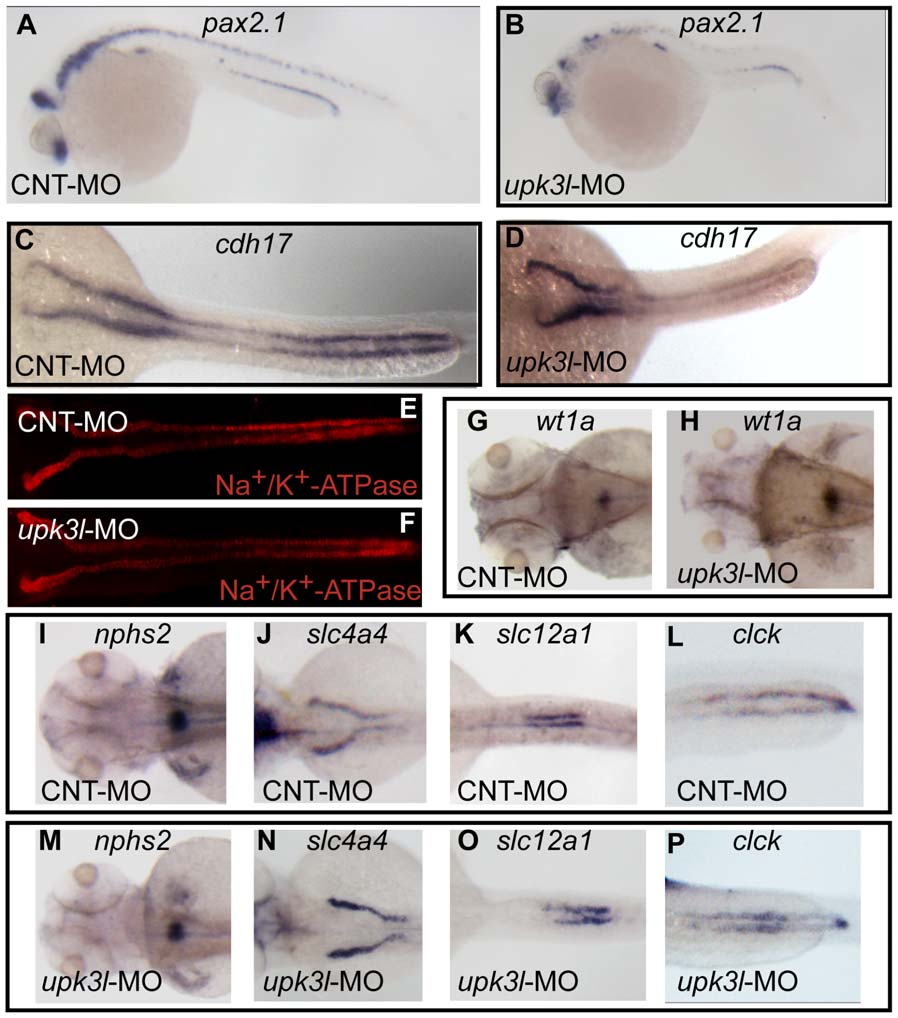
Pronephric morphogenesis, patterning, and segmentation in control and morphant embryos. (A–D) Expression of *pax2.1* in 1-dpf embryos or *cdh17* in 2-dpf embryos as revealed by *in situ* hybridization. (E, F) Low magnification overview of Na^+^/K^+^-ATPase expression in the PTs of 2-dpf control or morphant embryos. (G–P) Expression of *wt1a* (G–H), *nph2s* (I, M), *slc4a4* (J,N), *slc12a* (K, O) or *clck* (L, P) in control or morphant 3-dpf embryos.

Small molecules present in the circulatory fluid of larvae are filtered by the foot processes of glomerular podocytes and then enter the PT. A fraction of the filtrate is endocytosed in the proximal portion of the PT, whereas the remainder is modified by tubular reabsorption and/or secretion prior to excretion through the cloaca [13], [14]. When relatively large 500 kDa FITC-dextran was injected into the circulatory system of embryos, it remained unfiltered and was not internalized by control or morphant PT epithelia (Fig. 3C). These data, coupled with normal expression of podocyte markers (Fig. 4), indicates that morphants likely have an intact podocyte barrier. In contrast, uptake of a small, filtered FITC-dextran (10 kDa) was observed in the proximal tubule cells of control embryos where it was found in vesicles just below the apical membranes of the cells (Fig. 3C). However, in morphants, uptake of FITC-dextran was sparse and only a few dextran-filled vesicles were observed (Fig. 3C).

While it is difficult to assign primacy, the lack of Upk3l expression in the heart and circulatory system, the timing and modest nature of the cardiac effects, and the reduced uptake of 10 kDa dextran in the PT cells makes it more likely that the primary defect in morphants was related to diminished pronephros function.

### *Upk3l* morphants undergo expected pronephric morphogenesis, patterning, and segmentation

We next determined if the altered kidney function could be ascribed to defects in pronephric development. We examined expression of the *pax2.1* gene, an early-stage marker of pronephros development [22], as well as genes expressed in a segmented manner along the renal corpuscle and PTs (Fig. 4). At 1 dpf, *pax2.1* localized to the PTs of controls and morphants (Fig. 4A–B). At 2 dpf, early PT development is complete and the two PTs are easily distinguished by examining the distribution of *cdh17*, an adherens junction-associated protein expressed by the epithelial cells forming the PTs [23]. Morphants had a similar expression profile to control larvae (compare Fig. 4C to 4D). In addition, 3 dpf embryos immunolabeled with an antibody to the Na^+^/K^+^-ATPase, which labels the basolateral surfaces of PT epithelial cells [13], showed a grossly similar pattern of expression in controls and morphants (Fig. 4E–F). Furthermore, *wt1a*, a transcription factor that regulates early specification of podocytes [24], showed typical localization at the site of the glomeruli in both 3 dpf control and morphant embryos (Fig. 4G–H). In addition, the glomerular podocyte marker *nphs2* (podocin) [25], the proximal convoluted tubule sodium bicarbonate cotransporter *slc4a4*, the distal early tubule sodium/potassium/chloride transporter *slc12a1*, and the distal early/distal late/pronephric duct-associated *clck* chloride conductance channel all showed a similar pattern of expression in controls and morphants (Fig. 4I–P). Finally, a careful examination of the latter marker allowed us to confirm that the pronephric duct ended in the cloaca, which was contiguous with the external surface of the embryos. Taken together, these studies indicated that pronephric kidney morphogenesis, segmentation, and patterning were not substantially altered in morphants.

### Loss of Upk3l alters the polarization and morphogenesis of PT epithelial cells

**Figure 5:**
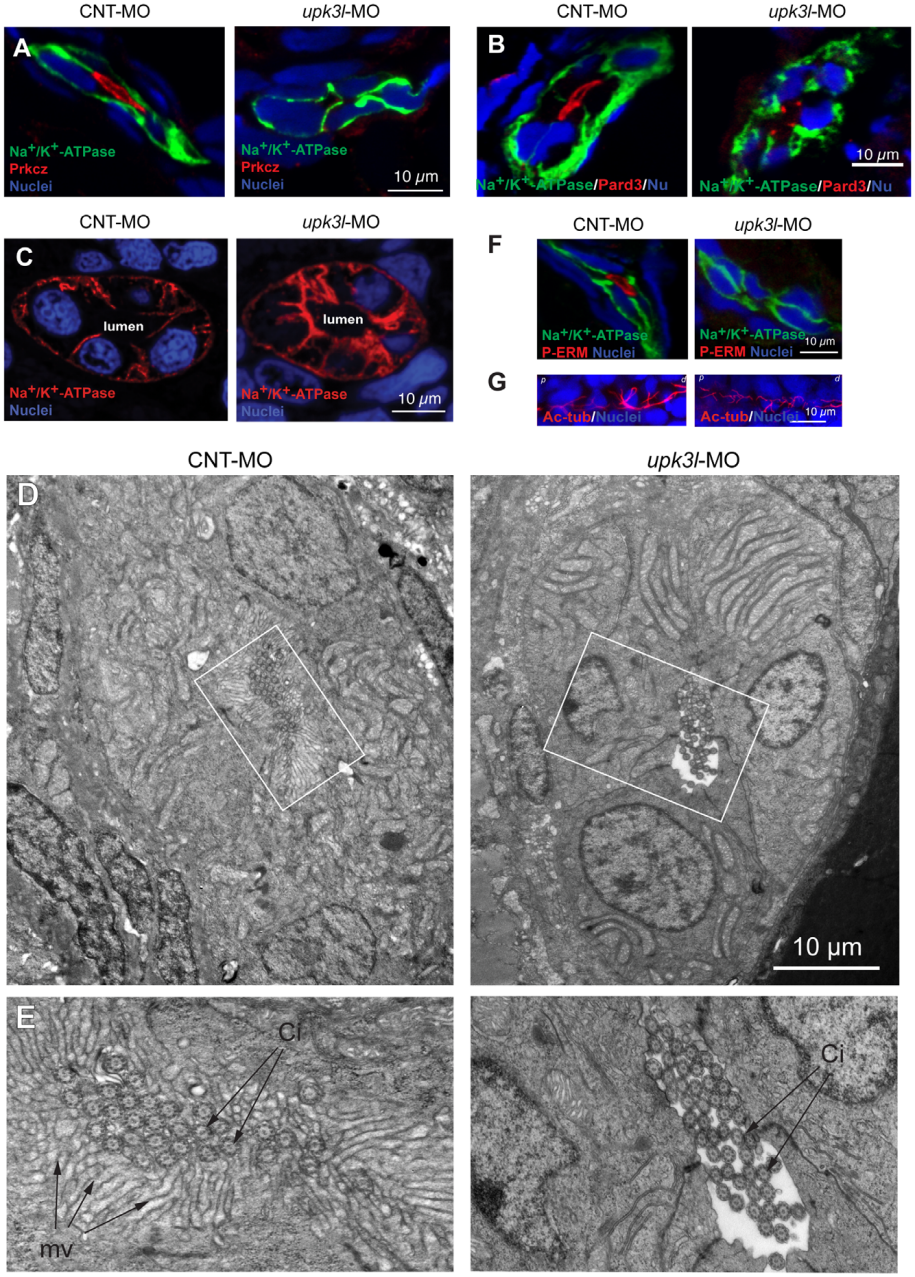
Disruption of epithelial polarity in *upk3l* morphants. (A–B) Localization of Prkcz (A) or Pard3 (B), Na^+^/K^+^-ATPase, and nuclei in cross-sectioned embryos treated with control (CNT-MO) or *upk3l* MO (*upk3l*-MO). (C) Distribution of Na^+^/K^+^-ATPase and nuclei in 2-dpf control or morphant embryos. (D–E) TEM analysis of the proximal segment of PTs from control or morphant embryos. (D) Cross section of tubule show that it is comprised of 5–6 cells surrounding a central lumen. (E) At higher magnification, control lumens were filled with microvilli (mv) and cilia (ci), whereas microvilli were absent in morphants. (F) Immunolocalization of phosphorylated ERM proteins (P-ERM) in frozen sections of embryos. Sections were co-stained for Na^+^/K^+^-ATPase and nuclei. (G) Immunostaining of cilia-associated acetylated-tubulin and nuclei in the PT of laterally oriented embryos. p: proximal tubule, d: distal tubule.

The formation of distinct apical and basolateral membrane domains are critical for epithelial functions such as vectorial transport of ions, solutes, and water [16]. We examined the distribution of the Par apical polarity complex, an assemblage of three proteins that is required for the formation of junctions and the apical membrane domain: aPKC, Par3, and Par6 [26]. Prkcz (aPKCζ is normally expressed at the apical surface and junctions of the PT cells (Fig. 5A) [27], [28]. However, this protein was absent from the apical pole of morphant PT epithelial cells (Fig. 5A). Furthermore, Pard3 (Par3) was distributed at the apical surface of control PT cells, but was found restricted to the apicolateral junctional complex in morphants (Fig. 5B). Whereas the overall segmental distribution of the Na^+^/K^+^-ATPase appeared normal in morphants (Fig. 4F), cross sections of PTs examined at higher magnification revealed that the Na^+^/K^+^-ATPase was mis-localized to the apical surface of some cells lining the PTs of morphants (Fig. 1F and 5C).

Upon polarization, completion of epithelial cell morphogenesis often includes the formation of specialized apical membrane domains including microvilli and cilia. Whereas the former serves several functions including amplification of apical surface area (>20-fold) [29], the latter are required for proper renal clearance [15]. To examine the morphogenesis of PTs, ultrathin sections of larvae were examined by transmission electron microscopy. This analysis revealed that in cross section the PT was comprised of 5–6 epithelial cells, whose apical surfaces formed the central lumen (Fig. 5D). In the more proximal regions of the pronephros, the apical surface of the principal cells was covered by an elaborate microvillar brush border, which extended into the lumen (Fig. 5E). Furthermore, multiciliated cells, which are prominent in the anterior portions of the pronephros [15], projected 9+2 motile cilia into the lumen (Fig. 5E). While the overall organization of the PT was similar in morphants (Fig. 5D), there was a striking loss of apical microvilli in the principal cells lining the PTs, leading to a “clear lumen” phenotype (Fig. 5E). In many regions the loss was complete (e.g. Fig. 5D); however, occasional microvilli were noted in other sites along the PT and an incomplete ring of actin was associated with these regions (Fig. 3C). The proper development of the brush border requires phosphorylated ERM proteins, including ezrin [30], [31]. Notably, we observed there was a lack of phosphorylated ERM proteins at the apical pole of morphant PT cells (Fig. 5F). Although cilia were still found in the lumens of morphant PTs, they appeared shorter and somewhat kinked when examined grossly by immunofluorescence (Fig. 5G). However, there were no obvious structural defects when these cilia were examined at the ultrastructural level (Fig. 5E). Taken together, these observations indicate that the *upk3l* morphant phenotype likely results from defects in the polarized distribution of proteins and brush border morphogenesis in PT epithelial cells.

### Specific residues in the Upk3l CT-domain regulate its function

**Figure 6:**
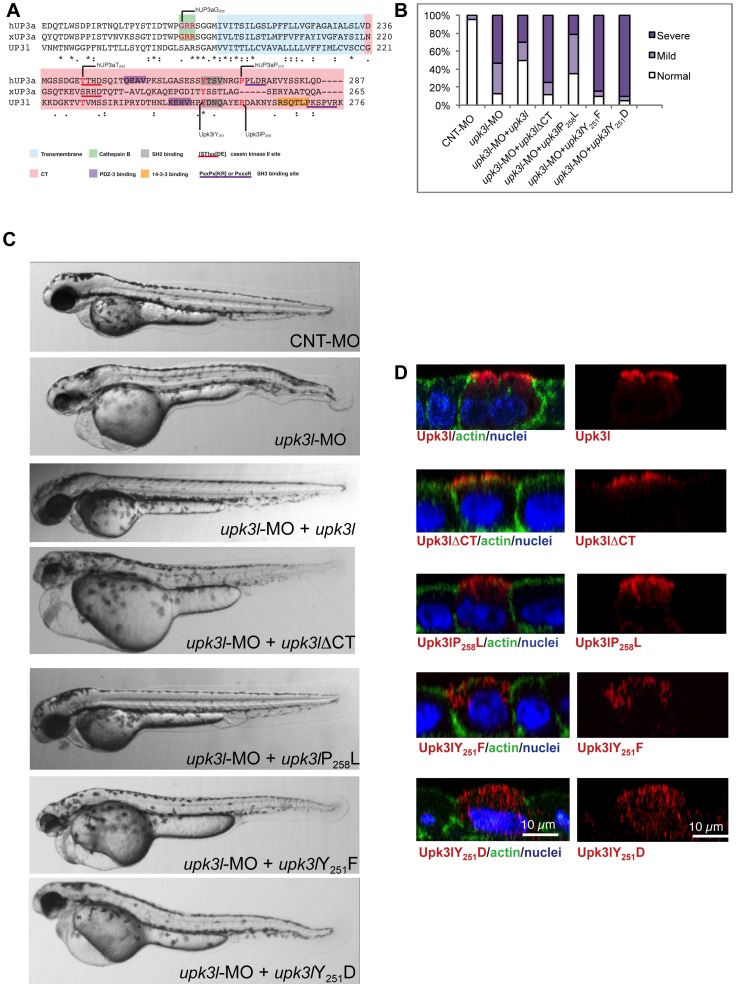
The CT of Upk3l is critical for its function. (A) Clustal W alignment of the TM and CT region of hUP3a, xUP3a and Upk3l. The amino acids that comprise the TM and CT domain are highlighted, and conserved functional motifs and residues are underlined or shaded, respectively. (B) Phenotypes of embryos injected with CNT-MO, *upk3l*-MO alone, or *upk3l*-MO co-injected with mCherry mRNA and mRNAs encoding MO-resistant versions of *upk3l*ΔCT, *upk3l*P_258_L, *upk3l*Y_251_F, or *upk3l*Y_251_D. Microinjection of MO-resistant mRNA alone at the same doses did not produce detectable phenotypic abnormalities. (C) Morphological phenotypes associated with embryos injected with 5 ng CNT-MO (*n* = 100), 3 ng of *upk3l*-MO (*n* = 100), or 3 ng of *upk3l*-MO and 100 pg of *upk3l*, *upk3l*ΔCT, *upk3l*P_258_L, *upk3l*Y_251_F, or *upk3l*Y_251_D mRNA (n≥50). (D) Localization of FLAG-tagged Upk3l, Upk3lΔCT, Upk3lP_258_L, Upk3lY_251_F, or Upk3lY_251_D in MDCK cells co-stained for actin and nuclei. Upk3lY_251_F and Upk3lY_251_D showed a predominantly intracellular localization and their low levels of expression necessitated an 1.5-fold increase in photomultiplier voltage above that used for the other samples.

Previous studies have identified motifs in the CT and membrane proximal domains of UP3a that are important for its function [8], [10], [11], [12]. Critical residues include a conserved proline residue that when mutated in humans (hUP3aP_273_L) leads to pronounced kidney defects [8]. This residue is present in Upk3l (P_258_), but absent in the *Xenopus* homolog xUP3a (Fig. 6A). Additionally, there is a conserved tyrosine residue (Y_251_ in Upk3l), which is critical for *Xenopus* egg activation but of unknown function in other species (Figure 6A) [10], [11]. We initially determined whether the CT domain was important for Upk3l function. Whereas full length Upk3l partially rescued the morphant phenotype, a construct lacking the CT domain (Upk3lΔCT) was unable to do so (Fig. 6B–C). We sought to confirm that the lack of rescue by Upk3lΔCT was not simply the result of say misfolding, which could lead to increased degradation or loss of surface delivery. Because the antibodies to Upk3l we generated no longer recognized this construct, we expressed FLAG-Upk3lΔCT in MDCK cells where it was targeted to the apical pole of the cell with little intracellular accumulation (Fig. 6D). Next, we examined the Upk3lP_258_L mutant, corresponding to human (h)UP3aP_273_L, which when co-expressed with hUP1b in COS-1 cells is found at the cell surface [8]. This variant was able to partially rescue the morphant phenotype, but not to the same extent as the full-length protein (Fig. 6B–C). While a fraction of this mutant was at or near the apical surface, some of it appeared to be present in vesicles in the subapical and lateral regions of the cell (Fig. 6D). A mutant that may act by preventing phosphorylation of residue Y_251_ (Upk3lY_251_F) failed to rescue, and a similar phenotype was observed for Upk3lY_251_D, a potentially phosphomimetic variant. Both of these mutants were expressed at low levels (the gain in Fig. 6D was increased 1.5-fold to visualize the signals) and were associated with intracellular vesicular elements. These elements were somewhat subapical in Upk3lY_251_F, but were more scattered in Upk3lY_251_D. Taken together, these results indicate that the CT of Upk3l contains motifs important for its function and that P_258_ and Y_251_ may act, in part, by regulating Upk3l stability and surface expression.

## Discussion

Why mutations in UPs lead to kidney malformation is not well understood, even though genetic analyses in patients and studies in UP3a and UP2 KO mice support a general role for UPs in urinary tract development and function [5], [6], [32]. To gain further insight into UP3a function, we analyzed the role of Upk3l in pronephros development. Our studies show that Upk3l is expressed at the apical surface of PT epithelial cells and plays an important role in epithelial polarization and morphogenesis. Data in support of this conclusion comes from analysis of morphant PT cells, which show a loss of Prkcz expression, altered Pard3 distribution, a lack of subapical phosphorylated ERM proteins, and a failure to generate an apical brush border membrane domain. An important consequence of these defects would be a significant decrease in apical surface area and a reduction of proteins that are stabilized by the subapical actin cytoskeletal network. An example of the latter may include the endocytosis machinery that normally promotes internalization of filtered fluids by proximal PT epithelial cells and is diminished in morphants. An additional polarity defect observed in morphants is apical expression of the Na^+^/K^+^-ATPase, which would likely impair the ability of PT epithelial cells to form the necessary electrochemical gradients needed to drive the flow of water into the PT lumen and recovery of ions across the PT epithelial cells. Abnormalities in electrolyte reabsorption could lead to the edema we noted in morphant embryos. Furthermore, the Na^+^/K^+^-ATPase also promotes adherens and tight junction formation, which in the heart requires interactions between the pump and the protein Nagie oko/Mpp5a (the zebrafish ortholog of the apical polarity complex protein Pals1/Stardust) [33]. Finally, it is possible that the altered luminal environment of the morphant PTs could negatively impact the function of the apical cilia, whose beating is necessary for pronephric clearance [15]. While we cannot rule out a role for altered glomerular function, or a defect in the innervation of the pronephros, our data point to a defect in PT epithelial polarity and morphogenesis as an important cause of the morphant phenotype.

Altered epithelial polarity and morphogenesis may also underlie the defects observed in those patients with renal adysplasia resulting from faulty UP3a expression. Kidney development in higher vertebrates proceeds in three stages, each one dependent on the function of the other: pronephros, mesonephros, and metanephros. The pronephros forms the first tubule in the kidney called the pronephric duct, which grows caudally and promotes differentiation of the surrounding mesoderm into mesonephric tubules. These latter elements drain into a continuation of the pronephric duct called the mesophephric duct, which eventually forms a pouch called the ureteric bud. This bud induces the surrounding mesenchyme to form the nephrons, while the bud tissue forms the collecting duct and ureter. Importantly, a defect in pronephric or mesonephric tubule polarity would likely negatively impact signaling between the epithelial cells and surrounding mesoderm, leading to altered kidney formation and adysplasia. Similarly, there is evidence that the ureteric epithelia, which is UP3a positive [8], signals through sonic hedgehog to the adjacent mesenchymal cells, promoting their differentiation into smooth muscle cells [34]. A failure of polarity development in the epithelia could lead to improper ureteric myogenic contractions. In turn, this could lead to a functional obstruction, the consequence of which includes kidney malformation [35].

Defects in epithelial polarity and morphogenesis may also explain some of the pathologies observed in the lower urinary tract of UP KO mice. For example, the umbrella cells of UP3a/UP2 KO mice appear undifferentiated: these cells lack apical surface plaques and their cytoplasms are filled with a large pool of immature fusiform vesicles, which normally delivery lipid and protein cargo to the apical cell surface [5], [6], [36]. The failure to sort the appropriate proteins/lipids into these vesicles, the inability to target these vesicles apically, or the lack of ability to fuse could explain, in part, why the uroepithelium of KO mice show increased permeability to urea, water, and dyes that are normally membrane impermeable [5], [6]. In addition, disrupted cell adhesion formation or turnover could explain why the umbrella cells of UP KO animals are hyperproliferative and occlude the lumens of ureters, which would promote vesicoureteral reflux [5], [36], and could secondarily alter kidney function and development [3].

How UP3 promotes epithelial polarization and morphogenesis is unknown, but is likely the result of interactions between its CT domain and signaling and/or polarity proteins. This possibility is consistent with our observation that Upk3lΔCT is unable to rescue the *upk3l* morphant phenotype, as well as reports that interactions between xUP3a and Src are critical for fertilized egg activation [10], [11], the discovery that bacterial-induced phosphorylation of hUP3a Thr_244_ by casein kinase II promotes umbrella cell apoptosis [12], and data showing that some patients with renal adysplasia have defects in the conserved GRR motif or a critical proline residue (P_273_) in the CT of hUP3a [8]. Intriguingly, Upk3l containing a similar mutation (Upk3lP_258_L) can only partially rescue the morphant phenotype. Furthermore, we observed that mutations in a conserved tyrosine residue, Y_251_, also had a dramatic impact on Upk3l function, apparently by altering the stability and apical trafficking of this protein. While this may be a simple effect of malfolding, it is equally plausible that a cycle of phosphorylation and dephosphorylation is critical for Upk3l apical delivery, turnover, and function. Interestingly, the ERM protein ezrin and aPKC, isoforms of which phosphorylate ezrin [37], may be important in the formation of the microvillar brush border. Thus, one role for Upk3l may be to promote these interactions. In this regard it is worth noting that UP3a contains SH2 and PDZ-3 binding motifs, which may engender interactions with polarity complexes (e.g. the Par complex), or other proteins that contribute to polarization including those associated with the adherens junction, the cytoskeleton, the brush border, or with membrane trafficking events. The identification of UP3a binding partners and further examination of the timing and sites of these interactions should provide important insight into how UP3a promotes urinary tract development in higher vertebrates.

## Materials and Methods

### Reagents

Unless specified otherwise, all chemical reagents were reagent grade or better and obtained from Sigma (St. Louis, MO). The α6F monoclonal antibody supernatant to the Na^+^/K^+^-ATPase was obtained from the Developmental Studies Hybridoma Bank (Iowa City, IA) and used at a 1∶50 dilution. The rabbit anti-PKCζ antibody was obtained from Santa Cruz Biotechnology (Santa Cruz, CA) and used at 1∶100 dilution. The rabbit anti-Par3 polyclonal antibody was purchased from EMD Millipore (Billerica, MA). The rabbit polyclonal antibody against phosphorylated-ezrin(T_567_)/radixin(T_564_)/moesin(T_558_) was obtained from Cell Signaling Technology (USA) and used at 1∶50 dilution. The mouse anti-acetylated tubulin monoclonal antibody (clone 6-11B-1) was obtained from Sigma and used at 1∶100 dilution. An anti-Upk3l rabbit polyclonal antibody was generated by Pickcell Laboratories (Amsterdam, UK). It was affinity-purified from the immune serum of rabbits injected with a peptide corresponding to residues 245–261 of the Upk3l cytoplasmic domain. The affinity-purified antibody was used at 0.18 mg/ml or further concentrated using Centricon Plus-70 Centrifugal Filter Units (Millipore). Mouse monoclonal anti-hUP3a antibody (K8B12) was used at 1∶20 dilution. Mouse FLAG-M2 (Sigma) and rabbit anti-HA antibodies (Covance; Emeryville, CA) were used at 1∶100 dilutions. Cy3/Dylight549-conjugated goat anti-mouse secondary antibodies (Jackson ImmunoResearch; West Grove, PA) were used at 1∶2500 dilution and FITC/Dylight488-conjugated goat anti-mouse and rabbit secondary antibodies (Jackson Immunoresearch) were used at a 1∶100 dilution. TO-PRO3, used at a 1∶1000 dilution, was obtained from Invitrogen (Carlsbad, CA). A 1∶20 dilution of anti-Upk3l or 1∶100 dilution of 6-11B-1 antibodies were used for western blotting.

### Zebrafish husbandry

The AB strain was maintained under standard conditions at the University of Pittsburgh zebrafish facility. Embryos were collected from group matings of wild-type AB adults. The embryos expressing the transgene Tg(*fli-1:EGFP*), kindly provided by Dr. Beth Roman (University of Pittsburgh), were obtained by mating heterozygous adults.

### Ethics statement

All studies were performed with the approval of the University of Pittsburgh Institutional Animal Care and Use Committee (IACUC).

### RT-PCR, bioinformatics, and whole-mount *in situ* hybridization

Whole genomic mRNA was extracted from 1- or 2- dpf embryos using TRI reagent (Ambion, Austin, TX) and the RNeasy Micro kit (QIAGEN, Valencia, CA). RT-PCR was performed using the following primers: forward - 5′-GAACACACACAATGCGATCCG-3′ and reverse: 5′-GATGAGATGTTCACTAGAGC-3′. The PCR product was sequenced by Genewiz DNA sequencing services (South Plainfield, NJ) and confirmed to be *upk3l* using Clustalw multiple sequence alignment software (http://www.genome.jp/tools/clustalw/). The predicted domains of Upk3l were identified using Prediction of Transmembrane Helices in Proteins software (http://www.cbs.dtu.dk/services/TMHMM/). A T7 promoter was attached to the 3′ end of *upk3l*-sequence using the following primers: forward - 5′-ATGAACACACACAATGCGATC-3′ and reverse - 5′-AATACGACTCACTATAGGGAGACGGGGGATGCGGATGGAGCTC-3′ (the T7 promoter sequence is underlined). DIG-labeled riboprobe was transcribed from the PCR product using T7 RNA polymerase and the DIG RNA labeling kit (Roche, Indianapolis, IN). Embryos were incubated in E3 medium (5 mM NaCl, 0.17 mM KCl, 0.33 mM CaCl_2_, 0.33 mM MgSO_4_) containing 0.003% (w/v) 1-phenyl-2-thiourea to suppress pigmentation. *In situ* hybridization was performed as described previously [38].

### Western blotting

For Western blotting, 2-dpf embryos were anaesthetized in E3 medium containing 0.2 mg/ml 3-amino benzoic acid ethyl ester (Tricaine), manually de-yolked with tweezers, and homogenized in 2× SDS Laemmli sample buffer. Samples containing equal amount of protein were resolved by SDS/PAGE and Western blots were performed as described previously [39].

### Embedding, fixation, sectioning, immunofluorescence labeling, and image capture

To label cryosections with anti-Upk3l and -α6F antibodies, the embryos were fixed overnight in Dent's fixative (80% v/v methanol, 20% v/v DMSO) at room temperature, and then stored in methanol at −20°C. Prior to cryo-embedding, the fixed larvae were incubated in 30% (w/v) sucrose in PBS overnight. The next day, embryos were transferred to Tissue-Tek® OCT compound (Sakura, USA), frozen in molds using ethanol-containing dry ice, and then sectioned (5 µm) using a CM1900 cryotome (Leica Microsystems, Buffalo Grove, IL). The sections were collected on Superfrost Plus microscope slides (Fisher Scientific, Pittsburgh, PA) and immunostained with appropriate dilutions of primary and secondary antibodies. Sections were post-fixed in 4% PFA, mounted and analyzed using confocal microscopy. Whole-mount immunostaining with α6F and 6-11B-1 was performed on 2dpf/3pf embryos fixed with Dent's fixative as previously described [16], [40]. To analyze α6F staining in cross-sections, the embryos were post-fixed in 4% PFA, embedded in JB-4 resin (Polysciences, Inc) as per the manufacturer's instructions, and then sectioned at 5 µm using a Leica ULTRACUT R microtome (Leica Microsystems).

MDCK cells, cultured on 12-mm, 0.4-µm Transwells (Costar, Cambridge, MA), were fixed 3-d post transfection with 4% (wt/vol) paraformaldehyde using pH-shift protocol [41], [42]. After fixation samples were quenched with phosphate-buffered saline (PBS) containing 20 mM glycine, pH 8.0, and 75 mM NH_4_Cl for 10 min at room temperature. Fixed cells were incubated with block buffer (0.025% [wt/vol] saponin, and 8.5 mg/ml fish skin gelatin in PBS) containing 10% (vol/vol) goat serum for 10 min at room temperature. Cells were incubated with primary antibody overnight at 4°C. Samples were washed three times with block buffer for 5 min, and then incubated with fluorescent-labeled secondary antibodies for 1 h at room temperature. After three additional 5-min washes with block buffer, the cells were rinsed with PBS, fixed with 4% paraformaldehyde for 5 min at room temperature and then mounted [42].

For fluorescently labeled samples, images were acquired using a 40× (N.A. = 1.25−0.75) oil CS objective and the appropriate laser lines of a Leica TCS SP5 CW-STED confocal microscope (in normal confocal mode) with the following parameters: photomultipliers were set at 600–900, and zoom at 12× and averaged eight times. Serial 0.3 µm z-sections were acquired and then reconstructed and analyzed using Volocity 4-D software (Perkin Elmers; Waltham, MA). Images were exported as TIFF files. When imaging intact embryos, they were maintained in egg water and oriented on Mat-Tek dishes (Ashland, MA). Alternatively, they were placed in glycerol on depression slides. Image acquisition was performed using a M125 dissecting stereomicroscope (Leica Microsystems) outfitted for epifluorescence, and equipped with a Fast1394 digital camera (QICAM, Surrey, Canada). Images were exported as TIFF files, contrast corrected in Photoshop CS5 (Adobe; San Jose, CA), and then imported into Adobe Illustrator CS5 where the final figure layouts were generated.

### Expression of Upk3l/UP3a in MDCK cells and injection of zebrafish embryos with MOs and mRNA

MDCK cells were transfected in suspension with the appropriate vector using Lipofectamine 2000 reagent (Invitrogen, Carlsbad, CA) and then cultured on 12- mm, 0.4-µm Transwells (Costar, Cambridge, MA). To generate zebrafish morphants, a translation-blocking antisense MO against the *upk3l* translation initiation site (*upk3l*-MO sequence: 5′- AACGGATCGCATTGTGTGTGTTCAT -3′) as well as CNT-MO were purchased from Gene Tools LLC (Philomath, OR). CNT-MO is a MO-Standard Control oligo (5′-CCTCTTACCTCAGTTACAATTTATA -3′). The MOs were injected in sterile ddH_2_O into the yolk of 1–2-cell embryos using an MPPI-2 microinjector (Applied Scientific Instrumentation, Eugene, OR). mRNA rescue experiments were performed using wild-type *upk3l* cDNA carrying five silent mutations within the *upk3l*-MO-targeted region (N2; AAC→AAT, T3; ACA→ACG, N5; AAT→AAC, I7; ATC→ATA, R8; CGT→CGA). The rescue construct was generated by RT-PCR based amplification of full-length *upk3l* from genomic mRNA extracted from 2-dpf embryos and its insertion between the *EcoR*I and *Xho*I sites of the pCS2(+) vector. Point mutations were introduced using the QuikChange mutagenesis kit (Agilent Technologies, Santa Clara, CA). Plasmid was linearized by *Not*1 digestion and capped MO-resistant *upk3l* mRNA was transcribed using the mMESSAGE mMACHINE SP6 kit (Ambion, Austin, TX) according to the manufacturer's protocol. mRNA for mCherry was transcribed from pCS2(+)-mCherry linearized with *Sac*II. mCherry mRNA (50 pg) was mixed with 50–200 pg *upk3l* mRNA in sterile ddH_2_0 and co-injected into 1-cell stage embryos prior to injections with *upk3l*-MO. Injected embryos were incubated in E3 medium (5 mM NaCl, 0.17 mM KCl, 0.33 mM CaCl_2_, 0.33 mM MgSO_4_) at 28°C. Embryos were observed using a dissecting stereomicroscope and images captured as described above.

### Clearance and dextran uptake assays

Solutions (1 mg/ml) of lysine-fixable Rhodamine-dextran (M_r_ 70,000; Invitrogen) or lysine-fixable fluorescein-dextran (M_r_ 10,000 and 500,000; Invitrogen) were prepared in 1× PBS. The tracers (1 ng) were injected into the common cardinal vein of 54-hpf embryos anaesthetized with 0.2 mg/ml Tricaine dissolved in E3 medium. For analyzing clearance, the injected embryos were transferred to individual wells of a 24-well plate at 28°C and then imaged individually 1, 5 and 24 h later. During image capture of injected larvae, the exposure time and gain were kept constant. The average fluorescence intensity of a fixed area in the center of the cardiac area was selected using the elliptical lasso tool command and quantified using the histogram tool of Photoshop CS5 software. The values are reported in relative units. Uptake of dextran by PT epithelial cells was evaluated at 1–1.5 h after injection of different molecular weight dextrans. Embryos were fixed in 4% PFA overnight at 4°C, embedded in OCT compound and sectioned as described above, and then stained and analyzed using the confocal microscope.

### Measurement of heartbeat and edema development

Live, unanesthetized embryos were oriented on depression slides and observed under the stereoscope. Heartbeat was measured by counting the number of times the atrium contracted to pump blood into the ventricle in 30 sec using a stopwatch and reported as beats/min. For edema measurements, images of individual embryos were acquired and the nominal two-dimensional area projected by the pericardial region was outlined and quantified using Photoshop CS5.

### Electron microscopy

Tissue was fixed in 2.0% (v/v) glutaraldehyde and 2.0% (w/v) paraformaldehyde in 100 mM sodium cacodylate buffer, pH 7.4 containing 0.5 mM MgCl_2_ and 1 mM CaCl_2_ for 60 min. The samples were then osmicated 1–2 h with 1.0% (w/v) OsO_4_ in 100 mM cacodylate, washed several times with distilled water, and then *en block* stained overnight in 0.5% (w/v) aqueous uranyl acetate. Embryos were dehydrated in a graded series of ethanol, embedded in the epoxy resin LX-112 (Ladd, Burlington, VT), and sectioned with a diamond knife (Diatome, Fort Washington, PA). Sections, silver in color, were mounted on butvar-coated nickel slot grids, contrasted with uranyl acetate and lead citrate, and viewed at 80 kV in a JEOL (Tokyo, Japan) 100 CX transmission electron microscope. Images were acquired using an L9C Peltier-cooled TEM camera system (Scientific Instruments and Applications, Inc.; Duluth, GA). Digital images were imported into Adobe Lightroom and the processed using the brightness, contrast, and clarity controls. When contrast was too low, adjustments to the tone curve were made to the whole image. Images were sharpened using a radius of 0.5 and luminance noise reduction performed. Composite images were generated using Illustrator CS5.
